# The Necrotic Signal Induced by Mycophenolic Acid Overcomes Apoptosis-Resistance in Tumor Cells

**DOI:** 10.1371/journal.pone.0005493

**Published:** 2009-05-11

**Authors:** Gwendaline Guidicelli, Benjamin Chaigne-Delalande, Marie-Sarah Dilhuydy, Benoît Pinson, Walid Mahfouf, Jean-Max Pasquet, François-Xavier Mahon, Philippe Pourquier, Jean-François Moreau, Patrick Legembre

**Affiliations:** 1 CNRS UMR 5164, Bordeaux, France; 2 Université Bordeaux-2, Bordeaux, France; 3 IFR-66, Bordeaux, France; 4 Centre Hospitalier Universitaire de Bordeaux, Bordeaux, France; 5 Service des maladies du sang, Hôpital du Haut-Lévêque, Pessac, France; 6 CNRS UMR 5095, Bordeaux, France; 7 INSERM U 876, Bordeaux, France; 8 INSERM E347, Institut Bergonié, Bordeaux, France; Mizoram University, India

## Abstract

**Background:**

The amount of inosine monophosphate dehydrogenase (IMPDH), a pivotal enzyme for the biosynthesis of the guanosine tri-phosphate (GTP), is frequently increased in tumor cells. The anti-viral agent ribavirin and the immunosuppressant mycophenolic acid (MPA) are potent inhibitors of IMPDH. We recently showed that IMPDH inhibition led to a necrotic signal requiring the activation of Cdc42.

**Methodology/Principal Findings:**

Herein, we strengthened the essential role played by this small GTPase in the necrotic signal by silencing Cdc42 and by the ectopic expression of a constitutive active mutant of Cdc42. Since resistance to apoptosis is an essential step for the tumorigenesis process, we next examined the effect of the MPA–mediated necrotic signal on different tumor cells demonstrating various mechanisms of resistance to apoptosis (Bcl2-, HSP70-, Lyn-, BCR-ABL–overexpressing cells). All tested cells remained sensitive to MPA–mediated necrotic signal. Furthermore, inhibition of IMPDH activity in Chronic Lymphocytic Leukemia cells was significantly more efficient at eliminating malignant cells than apoptotic inducers.

**Conclusions/Significance:**

These findings indicate that necrosis and apoptosis are split signals that share few if any common hub of signaling. In addition, the necrotic signaling pathway induced by depletion of the cellular amount of GTP/GDP would be of great interest to eliminate apoptotic-resistant tumor cells.

## Introduction

An increase rate in malignancies after organ transplantation is the toll to pay for allograft long-term survival and the post-transplant lymphoproliferation disorders (PTLD) represent the major cause of cancer-related mortality in kidney transplant recipients [1]. Mycophenolate mofetil (MMF) is an immunosuppressive agent widely used in transplantation the active compound of which, mycophenolic acid (MPA), depletes the intracellular pool of GTP through the inhibition of the inosine monophosphate dehydrogenase (IMPDH). IMPDH is found frequently over-expressed in tumor cells, making it an attractive target for the generation of anti-tumoral agents [2]. Recently it has been observed a tendency toward a lower risk of malignancy in the MMF versus non-MMF given transplanted patients [3] and MPA was endowed with an antitumoral action in an experimental in a *in vivo* tumor growth model [4].

Cell death plays an essential role in the homeostasis of tissues and organs and allows the elimination of infected or transformed cells. So far, three types of major cell death have been described: apoptotis (type I), autophagic cell death (type II) and necrosis (type III) [5]. Resistance to apoptosis occurs during tumorigenesis and explains tumor relapse following chemotherapeutic treatment. To evade apoptosis, tumor cells use various mechanisms, a number of which have not yet been characterized. For instance, chronic myeloid leukemia (CML) is characterized by the expression of a chimeric BCR-ABL oncoprotein in hematopoietic precursor cells [6] which behaves as a potent inhibitor of apoptosis [7]. Cancer cells from Chronic Lymphocytic Leukemia (CLL) are also reported to display a common default in apoptosis [8]. Furthermore, 80% to 90% of the low grade follicular non-Hodgkin lymphomas resist to apoptosis through the over-expression of Bcl-2 a potent inhibitor of the mitochondrion-dependent apoptotic signal [9].

Cdc42 is a key factor linking intracellular and extracellular signals to the organization of the actin cytoskeleton network [10]. This small GTPase belongs to the Rho-GTPase family. Herein, we demonstrated the pivotal role of Cdc42 to transmit the MPA-mediated necrotic signal. In addition, we explored whether this newly characterized necrotic signal shared common signaling hubs with various apoptotic pathways by examining the cytotoxic action of MPA on different tumor cells exhibiting resistance to apoptosis.

## Materials and Methods

### Ethics statement

All clinical investigation has been conducted according to the principles expressed in the Declaration of Helsinki. Blood was sampled from patients diagnosed with B-CLL after written consent was obtained from each individual. This study was approved by institutional review board at the Centre Hospitalier Universitaire de Bordeaux.

### Patients

All CLL patients were Binet stage A. Using Ficoll separation and elimination of monocytes by adherence, the purified B-lymphocytes (>85% of the isolated cells were CD19^+^CD5^+^ B lymphocytes) were maintained in a RPMI medium supplemented with 8% human serum.

### Cells

The lymphoblastoid B-cell lines Dab-1, the leukemic T-cell lines CEM and Jurkat and the chemotherapy-resistant cells were grown in RPMI 1640 supplemented with 8% v/v heat-inactivated FCS and 2 mM L-glutamine at 37°C in a humidified atmosphere containing 5% CO_2_. PBLs (peripheral blood lymphocytes) from healthy donors were isolated by Ficoll gradient centrifugation exactly as described previously [11]. Doxorubicin-resistant Jurkat and CEM cells were derived by treatment with stepwise increase in the concentration of doxorubicin and clones were isolated by the limiting dilution method. Generation of Bcl-2 over-expressing Jurkat cells was described elsewhere [12].

### Reagents

Guanosine, adenosine, ribavirin, mycophenolic acid, latrunculin A and cytochalasin D were purchased from Sigma (St Louis, MO, USA). Soluble CD95L was generated in the laboratory [13]. Nilotinib (AMN-107) was obtained by Dr Mahon F.X. from Novartis Pharma AG. The anti-Bcl-2 mAb and the anti-CD95 mAb (clone DX2) were purchased from Pharmingen (BD Biosciences, San Diego, CA, USA). Killer TRAIL was purchased from Alexis (Coger, France). Isotypic antibody (anti-LIF, clone 1F10) was a home-generated mAb. Secramine A was synthesized by Dr Xu B. and Hammon G.B. (University of Louisville, Louisville, USA) and was kindly provided by Dr Kirchhausen T. (Harvard Medical School, Boston, USA).

### Plasmids and transfection

Q61L-Cdc42-GFP and Q61L-Rac1-GFP were obtained from Dr Fort P. (Montpellier, France). Two small hairpin RNA (shRNA) targeting Cdc42 were designed (shRNA#3 5′-agactcctttcttgcttgttgg-3′ and shRNA#4 5′-agtatgtggagtgttctgcact-3′) and cloned into the plasmid pSuppressorNeo (Imgenex, San diego, CA, USA). A negative control plasmid with a scrambled sequence was supplied by Imgenex. The pEGFP-Bcl-2 plasmid was kindly provided by Dr Youle R. (NIH, Bethesda, MD).

Cells (5.10^6^ cells in 0.3 ml) were electroporated with 10 µg of DNA using a BTX ECM 830 electroporation generator (BTX Instrument Division, Holliston, MA, USA). Twenty hours later, living cells were isolated using a Ficoll separation method as described elsewhere [13].

For transient transfection assay, Jurkat T cells were electroporated with the GFP-fused constitutive active mutants of Cdc42 and Rac1 alone or the cells were co-transfected with the indicated shRNA and the pEGFP-N1 vector at a ratio of 3∶1 (10 µg of total DNA). After pulse discharge, the cells were placed in 6 ml of complete medium and incubated for 20 hours at 37°C. Living cells were isolated over a Ficoll gradient and then treated or untreated with MPA for 40 hours.

### Western blot analysis

Cells were lysed for 30 minutes at 4°C in lysis buffer (25 mM HEPES pH 7.4, 1% v/v Triton X-100, 150 mM NaCl, supplemented with a mix of protease and phosphatase inhibitors (Sigma)). Protein concentration was determined using the bicinchoninic acid method (Pierce, Brebières, France) according to the manufacturer's protocol. Proteins were separated by electrophoresis using a SDS-PAGE and transferred to a nitrocellulose membrane (Amersham Biosciences, Buckinghamshire, UK). Immunoblots were performed exactly as previously described [12].

### Phalloidin staining

Cells were pre-incubated with the inhibitors of actin polymerization for 30 min at 37°C, then fixed in PBS/2% PFA for 30 minutes, washed and treated for 15 minutes with PBS/5% FCS to quench aldehyde groups. Cells were permeabilized 5 min at 4°C using PBS/0.1% Triton X-100 and stained with FITC-linked phalloidin (Sigma) (1 µg/ml) for 30 min at 4°C. Cells were washed and the intensity of fluorescence was assessed by flow cytometry using the CANTO II flow cytometer (BD Biosciences).

### Cell death assays

Cell viability was assessed using the metabolic assay MTT, as previously described [14].

The necrotic morphology was assessed by flow cytometry as described previously [15]. Briefly flow cytometric parameters, cell size (FSC) and granule content of the cells (SSC), allowed to define a window corresponding to the living cells (untreated cells). MPA treatment revealed a population which harbored a size similar to living cells but contained more granules. This cell population remained insensitive to the inhibition of caspases using zVAD-fmk pre-treatment and corresponded to the necrotic cells [15].

To assess plasma membrane permeability, treated or untreated cells were incubated for 1 hour with regular medium containing the membrane-impermeant dye propidium iodide (20 µg/ml) and then immediately analyzed by flow cytometry.

Measure of the mitochondrial Potential (ΔΨm). Cells were pre-incubated with DiOC_6_ (10 nM) for 15 min and then stimulated with CD95L or MPA. Cell fluorescence was measured by flow cytometry.

### Dosage of intracellular GTP/GDP levels

Cellular extracts were prepared using an ethanol extraction method. Treated or untreated cells (10×10^6^ cells) were lysed (10 mM Hepes pH 7.2, 75% v/v Ethanol) at 80°C for 3 minutes. The ethanol/Hepes solution was evaporated in a rotavapor apparatus. The pellet was resuspended in 500 µl water and guanine-derivatives were separated by HPLC on a CarboPac PA10 column (250 mm×4 mm; Dionex Sunnyvale, CA, USA) equipped with PA1 guard column. Guanine derivatives were detected at 260 nm with a PDA100 photodiode array detector (Dionex) and were quantified by comparison with calibration curves obtained with guanine-derivative standards.

### Electronic microscopy

Treated cells (10^7^) were fixed for 1 hour at 4°C in 1.5% v/v glutaradehyde (0.1 M phosphate buffer), post-fixed in 1% w/v osmium tetroxide for 1 hour at 4°C, dehydrated in ethanol and embedded into a Epon resin mixture. Ultra-thin sections were double stained using uranyl acetate and lead citrate. Finally, sections were analyzed using a FEI Tecnai 12Biotwin (FEI, Hillsboro, OR, USA) transmission electron microscope.

## Results

### The GTP/GDP-depletion drives a Cdc42 and actin-dependent necrotic signal

**Figure 1:**
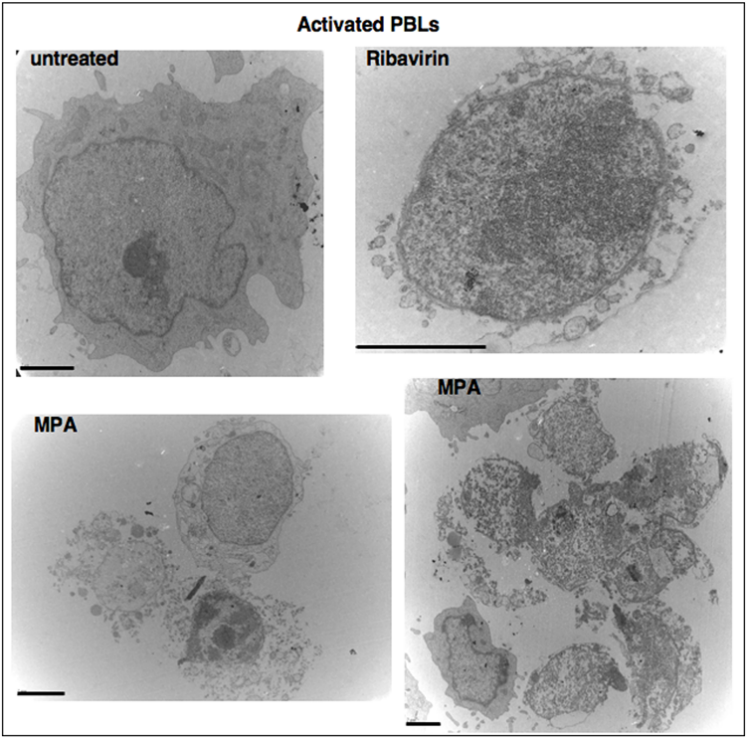
IMPDH inhibition leads to a necrotic morphology. Electron micrographs of activated PBLs. Cells were incubated with MPA (3 µg/mL) or with ribavirin (100 µg/ml) for 44 hours (Bars = 2 µm).

We previously showed that therapeutical doses of MPA and Ribavirin, two potent inhibitors of the IMPDH led to the elimination of activated PBLs through a caspase-independent necrotic signal [15]. Using transmission electron microscopy, we confirmed that activated PBLs were eliminated mainly by necrotis (Figure 1).

**Figure 2:**
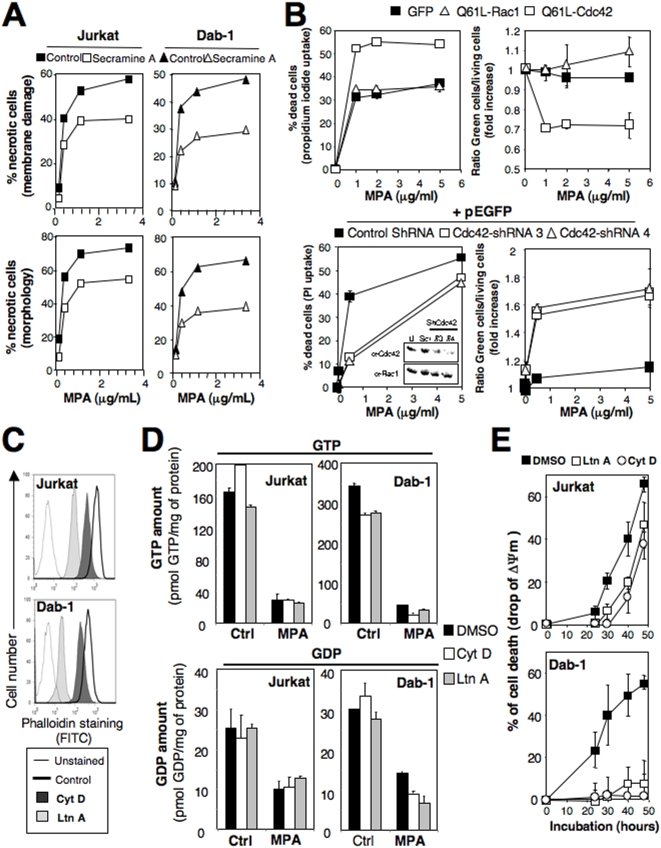
Cdc42 and actin are implicated in the MPA-mediated necrotic signal. (A) Cells were pre-incubated with secramine A (40 µM) or DMSO for 1 hour and then incubated for 44 hours with MPA. Necrosis was quantified by assessing the loss of the plasma membrane integrity (entry of propidium iodide) and the cell morphology. (B) *Upper panel:* Jurkat cells transfected with plasmid encoding Q61L-Cdc42-GFP, Q61L-Rac1-GFP or GFP alone were treated with MPA for 44 hours. The percentage of dead cells (morphologic analysis) and the percentage of GFP-expressing cells among the living population are shown. *Lower panel:* Jurkat cells were transfected with the Cdc42-targeting shRNA or control shRNA (Scr) and pEGFP-N1 at a DNA weight ratio of 3∶1. 24 hours after transfection, living cells were isolated by Ficoll gradient and incubated for 44 hours with MPA. Cell death was assessed by analyzing plasma membrane damage (propidium iodide entry). The inset depicts the expression of Cdc42 and Rac1 in Jurkat cells untransfected (U) or transfected with scramble shRNA (Scr) or shRNA targeting Cdc42. Western blots were performed using the indicated antibodies. (C) The B-cell line Dab-1 and the T-cell line Jurkat were treated with Latrunculin (LtnA, 2.5 µM), Cytochalasin D (CytD, 5 µM) or DMSO (control) for 30 minutes. Cells were then fixed, permeabilized and F-actin was tagged using FITC-labeled phalloidin. The amount of F-actin was assessed by flow cytometry. (D) Cells were pre-incubated for 30 min with 2.5 µM of LtnA, 5 µM of CytD or DMSO and then treated in presence or not of MPA (5 µg/ml) for 14 hours. The amount of GTP (*upper panel*) and GDP (*lower panel*) was quantified as described in Materials and Methods. (E) Cells were pre-incubated for 30 min with LtnA (2.5 µM) or CytD (5 µM) and then treated with 5 µg/ml of MPA for the indicated times. ΔΨm was assessed using DiOC6 staining. Data represent the mean±SD of three independently performed experiments.

We recently pointed out that MPA promoted the activity of Cdc42, which was important to transmit the necrotic signal [15]. To formally prove the involvement of Cdc42 in the necrotic signal, we tested the effect of a new selective inhibitor termed Secramine A [16]. Secramine A decreased the proportion of MPA-induced necrotic cell death in both the B cell line Dab-1 (40 to 50% inhibition) and in the T cell line Jurkat (40% inhibition) (Figure 2A) as assessed by the measurements of the cell morphology (flow cytometry analysis using FSC and SSC parameters, see [15]) or the plasma membrane permeability (cell entry of propidium iodide). Since GTPases switch from an active, GTP-bound, to an inactive, GDP-bound form, by the hydrolysis of GTP, we next examined the effect of a GTP-locked version of Cdc42 (constitutively active mutant termed Q61L-Cdc42) or of Rac1 (Q61L-Rac1 was taken as control) upon MPA-mediated cell death. The constitutive active GTPases were fused to GFP (green fluorescent protein). After transfection, living cells were isolated and the proportion of cells expressing the chimeric protein (green cells) was comprised between 30 and 40%. Unlike Q61L-Rac1 or GFP alone, the expression of the constitutive active mutant of Cdc42 significantly enhanced the necrotic signal generated by the addition of MPA (see Figure 2B, upper panel). The increased rate of cell death was correlated to a significant decrease of the ratio of green cells among the living cells (Figure 2B, upper panel) indicating that a constitutive active Cdc42 promoted the necrotic signal triggered by the addition of MPA. We next examined the effect of the Cdc42 silencing on the necrotic signal using small hairpin RNAs (shRNA). Since the small GTPases Rac1 and Cdc42 share 66.8% of DNA sequence identity, we controlled the specificity of the designed shRNA towards Cdc42 by comparing its action on the expression level of both Cdc42 and Rac1. Two different shRNA down-regulated the Cdc42 expression, whereas they did not modify the protein expression of Rac1 (Figure 2B, lower panel, see the inset). The T cell Jurkat was co-transfected with the shRNA and the GFP encoding plasmids at a molecular ratio of 3∶1 and we followed the fate of the green cells (GFP positive) upon the addition of MPA. For each condition, green cells corresponded to 40–60% of the living cells. As shown in Figure 2B (lower panel), the down-expression of Cdc42 dramatically impaired the MPA-mediated necrotic signal. Additionally, the silencing of Cdc42 was correlated with an enrichment of the GFP-expressing cells among the living cells (Figure 2B, lower panel). Taken together these results established that Cdc42 played a pivotal function in the transmission of the MPA-mediated necrotic signal.

By remodeling the actin cytoskeleton, Cdc42 participates in various cellular processes such as cell motility and cytokinesis [10]. To identify where actin remodeling acts in the molecular ordering of the MPA-mediated necrotic signal, we investigated the effect of inhibitors of actin polymerization on an early step (depletion of GTP and GDP) and a late step (loss of mitochondrial potential ΔΨ_m_), the latter being considered as an irreversible process in cell death [5]. As Phalloidin displays a strong affinity for polymerized actin (F-actin) [17], fluorescent-conjugated phalloidin was used to monitor by flow cytometry the amount of F-actin in permeabilized cells. Latrunculin A (LtnA) and cytochalasin D (CytD) are potent inhibitors of actin polymerization. The pre-incubation of the B-cell line Dab-1 and the T-cell line Jurkat with non-toxic concentrations of these chemicals significantly decreased the phalloidin staining (Figure 2C) confirming that actin polymerization was prevented in the treated cells. The dramatic decrease of the intracellular pools of GTP and GDP observed during the inhibition of the IMPDH (Figure 2D) remained unchanged when actin polymerization was abrogated either by latrunculin A or cytochalasin D (Figure 2D). Conversely, the inhibitors of actin polymerization significantly impaired the drop of ΔΨ_m_ induced by MPA in the T cell line Jurkat and completely abrogated it in the B cell line Dab-1 (Figure 2E). These findings established that the necrotic signal induced by MPA relied on the remodeling of the actin cytoskeleton and this cellular process took place downstream the GTP depletion and upstream the abrogation of mitochondria functions.

### Apoptosis-resistant tumor cell lines remain sensitive to the necrotic signal

Resistance of the malignant cells to apoptosis is an important issue in oncology, hence we next investigated whether the newly characterized necrotic signal remained efficient to eliminate *in vitro* and *ex vivo* tumor cells that harbored different molecular mechanisms to resist apoptosis.

**Figure 3:**
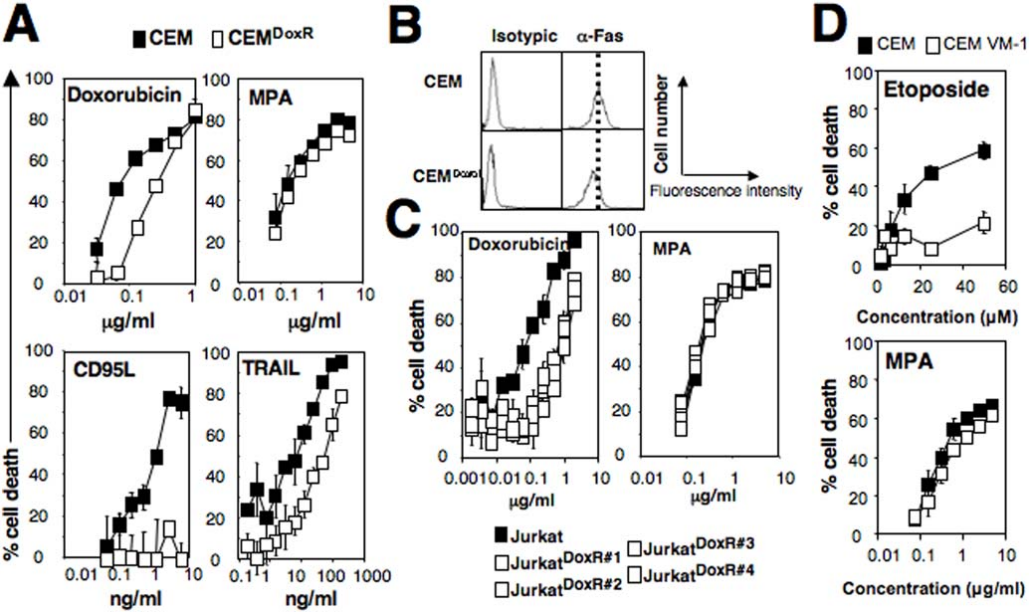
Apoptosis resistances are bypassed by the MPA–mediated necrotic signal. (A) Parental CEM and doxorubicin-resistant CEM (CEM^DoxR^) were incubated with indicated dose of doxorubicin, CD95L, TRAIL or MPA for 48 hours. Cell death was measured using a MTT assay. (B) Flow cytometry analysis of the CD95 expression on the doxorubicin-resistant CEM. (C) Parental Jurkat and the selected doxorubicin resistant clones (Jurkat^DoxR^) were incubated for 48 hours with doxorubicin and MPA at the indicated concentrations. MTT assay was performed to quantify cell death. (D) CEM and the etoposide resistant CEM-VM1 were incubated with the etoposide or MPA for 48 hours and cell death was assessed using MTT assay. Results shown represent the mean±SD of three independent experiments.

Doxorubicin, which is commonly used for the treatment of non-Hodgkin's lymphomas, induces an apoptotic signal [18]. From the leukemic T-cell lines Jurkat and CEM, we generated tumor cells resistant to doxorubicin-mediated apoptosis (Figure 3A and 3C). The selected clones did not display any modifications of the influx or efflux of doxorubicin indicating that the expression and the activity of the ATP-Binding Cassette, subfamily B (ABCB1/MDR-1) remained unchanged in these cells and therefore could not be held responsible for drug resistance (data not shown). Doxorubicin has been described to induce cell death of leukemia cells through the induction of a CD95L-dependent apoptotic signal [18]. As a consequence, it was expected that a selected doxorubicin-resistant CEM clone (CEM^DoxR^) was resistant to the apoptotic inducer CD95L (Figure 3A). This clone expressed a similar amount of membrane CD95 compared to its respective parental cell line (Figure 3B). Since the TRAIL-mediated apoptotic signal was also impaired in this CEM^DoxR^ (Figure 3A), we concluded that the default in apoptosis was not caused by a CD95 mutation but instead, by the modulation of a yet undefined intracellular target. It is noteworthy that compared to the parental cell line, none of the isolated Jurkat^DoxR^ clones exhibited resistance to CD95L (data not shown) suggesting that these clones displayed a different mechanism to resist doxorubicin-mediated apoptosis compared to the one present in the CEM^DoxR^ cell line. Despite their resistance to apoptosis, the magnitude of MPA-mediated cell death was not altered in the CEM^DoxR^ and Jurkat^DoxR^ compared to their respective parental cells (Figure 3A and 3C). The inhibitor of topoisomerase II etoposide is used for the treatment of refractory non-Hodgkin lymphomas [19]. Using the etoposide resistant cell line CEM VM-1 [20], we observed that it displayed a similar sensitivity to MPA compared to the parental cell line (Figure 3D). These data suggest that there does not exist any crosstalk between the necrotic signal triggered by MPA and different molecular mechanisms developed by cells to resist etoposide or doxorubicin-induced apoptotic cell death.

**Figure 4:**
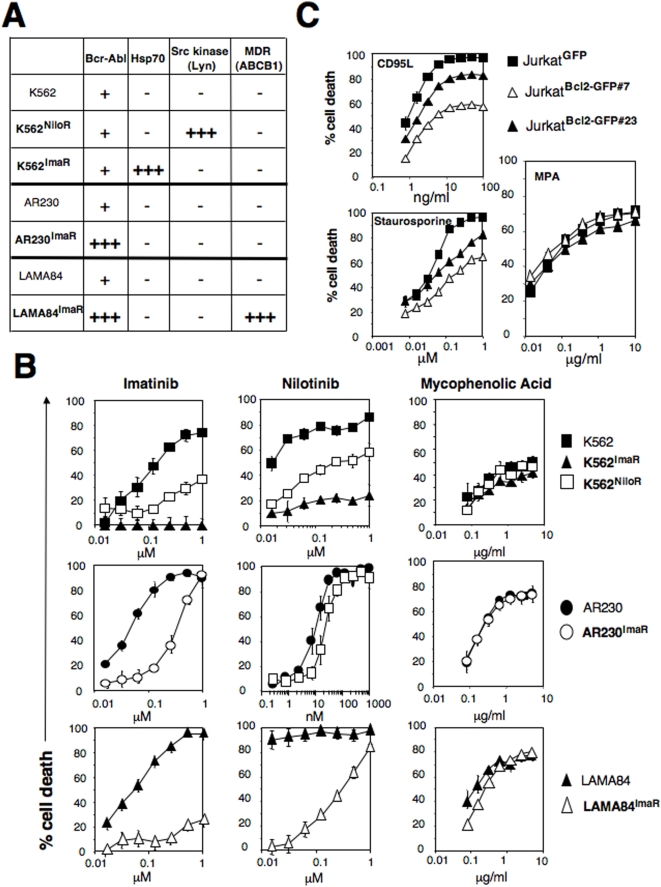
MPA mediates necrosis in malignant cells resistant to apoptosis. (A) Molecular characterization of the imatinib and nilotinib-resistant CML cell lines. MDR: multidrug-resistance related protein. (−) indicates an expression level similar to the untreated cells, (+) refers to the ectopic expression of BCR-Abl and (+++) indicates the over-expression of the indicated protein. (B) Imatinib or nilotinib-resistant CML cells were incubated with the indicated concentrations of imatinib (left panel), nilotinib (middle panel) or MPA (right panel) for 48 hours. Cell death was measured by MTT assay. (C) Expression of Bcl2 (endogenous and GFP-Bcl2) was assessed in GFP- and GFP-Bcl2-expressing clones by immunoblot. The indicated cells were incubated for 24 hours with soluble CD95L, staurosporine or MPA for 48 hours. Total cell death was measured by MTT.

Chronic myeloid leukemia (CML) cells express an oncogene protein consisting of the gene fusion of BCR (Breakpoint Cluster Region) and the tyrosine kinase ABL (Abelson), which behaves as a potent inhibitor of apoptosis [7]. Imatinib and nilotinib, two tyrosine kinase inhibitors, are used for the treatment of chronic myeloid leukemia suffering patients. By incubating different chronic myelogenous leukemia-derived cell lines (K562, AR230, LAMA84) with imatinib or nilotinib, resistant cells were selected. To resist cell death induced by the inhibitors of BCR-ABL activity, these cells promote different molecular mechanisms such as amplification of the *BCR-ABL* locus [21], over-expression of the members of src kinase family [22] or the increase expression of the anti-apoptotic chaperone protein Hsp70 [23] (Figure 4A). Hsp-70 has been reported as a potent inhibitor of apoptotic signals induced by lysosomal membrane permeabilization [24], death receptor signaling and ceramide production [25]. The over-expression of the kinase Lyn prevents the apoptotic signal triggered by tyrosine kinase inhibitors [26]. Although these different molecular mechanisms were very efficient to protect the CML cells from imatinib (Figure 4B, left panel) and nilotinib-mediated apoptotic signals (Figure 4B, middle panel), they turned out to be unable to cripple the necrotic signal induced by MPA (see Figure 4B, right panel).

The anti-apoptotic factor Bcl2 is over-expressed in various non-Hodgkin lymphomas in which it prevents cell death induced by numerous apoptotic inducers targeting the mitochondria [27]. We over-expressed Bcl-2 in the leukemic T-cell line Jurkat and we analyzed the effect of this potent anti-apoptotic factor upon the MPA-mediated necrotic signal. As expected, Bcl2 impinged on the deaths triggered by both the apoptotic inducers CD95L (extrinsic death inducer) and staurosporine (intrinsic death inducer), whereas it remained inefficient to alter the MPA-mediated cell death (Figure 4C).

Taken together these findings showed that the necrotic signal induced by the depletion of GTP/GDP did not share any common signaling hubs with the array of apoptotic pathways triggered by various chemotherapeutic agents.

### Tumoricid action of MPA towards primary tumor cells

Tumor cells isolated from Chronic Lymphocytic Leukemia (CLL) patients share the common feature to be resistant to apoptosis [28]. In addition, the isoform 2 of IMPDH has been found over-expressed in these tumoral B cells compared to normal lymphocytes [29]. TNF-Related Apoptosis-Inducing Ligand (TRAIL) is an apoptotic cytokine that exhibits a cytotoxic action on leukemic and lymphoma cells either alone or in combination with chemotherapeutic agents [30]. Similarly to TRAIL, CD95L belongs to the TNF (tumor necrosis factor) family and is a potent inducer of apoptosis. To determine whether MPA was able to achieve an anti-tumoral action on primary tumor cells resistant to apoptosis, we compared its effect to TRAIL and CD95L on freshly isolated B-CLL cells.

The concentration of TRAIL used to kill CLL (500 ng/ml) cells was comprised between the residual and the maximum of the TRAIL concentration measured in the sera of patients included in phase Ia and Ib clinical trials [31]. This concentration killed between 80 and 100% of the lung carcinoma (A427, H2122), colorectal adenocarcinoma (COLO 205, SW948, DLD-1), pancreas adenocarcinoma (BxPC3, HPAC) [32]. The concentration of CD95L (500 ng/ml) applied on the B-CLL cells eliminated 100% of the EBV-transformed lymphoid (SKW 6.4, Dab-1) and the T leukemia (Jurkat, CEM) cell lines (data not shown).

**Figure 5:**
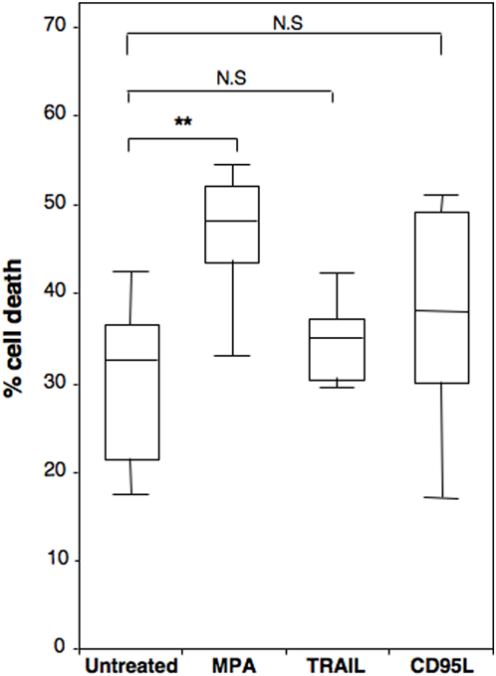
MPA treatment efficiently kills B-CLL cells. Leukemic B-cells isolated from CLL patients (n = 14) were treated with either the apoptotic inducers TRAIL (500 ng/ml) or CD95L (500 ng/ml) or with MPA (10 µg/ml) for 72 hours. Then total cell death was quantified using the metabolic assay MTT (total cell death). Statistical analysis was performed using a non-parametric Mann-Whitney two sample ranksum test (n = 14). N.S means non-significant and ** P≤0.01.

It is noteworthy that around 30% of the isolated B-CLL cells underwent spontaneous cell death (see untreated cells in Figure 5). Quantification of cell death revealed that untreated B-CLL cells were not significantly sensitive to TRAIL or CD95L (spontaneous cell death corresponded to 31.1±8.3 vs 35.6±4.6 for TRAIL and 38±11.2 for CD95L, Figure 5). In contrast these malignant cells underwent a significant and more potent cell death signal when incubated with therapeutical dose of MPA (10 µg/ml) (31.1±8.3 vs 45.6±8.8). Taken together, these findings ascertained that primary tumor cells reported to be highly resistant to apoptosis still remained sensitive to the MPA-mediated necrotic signal.

## Discussion

The cell-depletion of GTP/GDP eliminates cells through a necrotic signal that is reliant on intact activity of Cdc42 and the remodeling of actin. We show for the first time that the atypical and newly characterized necrotic signal triggered by the inhibition of the IMPDH activity remains unaffected in different tumor cell lines and in freshly isolated cancer cells for which the apoptotic signal is crippled.

Resistance to apoptosis is an essential step in the tumorigenesis process allowing the cell to increase its genetic instability and to escape from immunosurveillance. Leukemic and lymphoma cells exhibit the deregulation of a large range of cellular processes leading to escape the immune system and/or the chemotherapeutic treatments and thereby to overcome their tumoricidal action. In accordance with our findings, Dyer and colleagues argued that numerous tumor cells were resistant to TRAIL-mediated apoptosis [33] and the same group pointed out that the combination of TRAIL with histone deacetylase inhibitors was required to sensitize tumor cells to death [34]. In our hands no additional pre-treatment was necessary to sensitize CLL cells to the necrotic effect mediated by therapeutical dose of MPA. These findings suggest that the use of IMPDH inhibitors alone or in combination with other chemotherapeutic agents may promote the elimination of CLL cells. As a consequence, a more comprehensive knowledge of the necrotic signal mediated by the depletion of GTP/GDP would be of a great value to disclose new therapeutic targets with the purpose of eliminating apoptosis resistant tumors.

Numerous targets have been characterized for Cdc42 and the human Wiskott–Aldrich syndrome protein (WASP) is an effector of Cdc42 playing an important function in the remodeling of actin upon Cdc42 activation. The WASP family includes WASP, N-WASP (neural WASP), WAVE (WASP verprolin homologous)-1, WAVE-2 and WAVE-3. Mateo and colleagues showed that the antibody-mediated aggregation of the CD47 antigen leads to the induction of a caspase-independent signal responsible of the B-CLL death [35]. The authors also found that WASP-deficient cells were unable to transmit the non-apoptotic signal. Using wiskostatin as a selective inhibitor of N-WASP, we did not observe any modification of the MPA-mediated necrotic signal (Chaigne-Delalande B. and Legembre P., unpublished data) indicating that N-WASP was probably not the link between Cdc42 and actin polymerization. In the context of the MPA-mediated necrotic signal, it is conceivable that the actin remodeling occurs independently of WASP [10], even so we can not rule out that other members of the WASP family are involved upon the activation of Cdc42.

Atypical necrotic or necrotic-like signals have been recently described. However, whereas high doses of CD95L drove a necrotic signal relying on the expression of RIP and FADD [36], the caspase-independent signaling pathway induced by the insulin-like growth factor I receptor stimulation occurred through the activation of c-Jun N-terminal kinase (JNK) [37]. We previously showed that RIP and FADD deficient cells responded to MPA identically to the parental cells and inhibition of the JNK activity did not impinge on the cell death signal mediated by the IMPDH inhibition [15]. Furthermore, accumulation of the polyADP ribose polymers, the product of the enzyme PARP-1 (poly(ADP ribose) polymerase) has been reported as an inducer of necrosis upon incubation with the alkylating agent N-methyl-N'-nitro-N-nitrosoguanidine (MNNG) [38]. In fact MNNG induced a necrotic signal through the release from the mitochondria of the Apoptosis-Inducing Factor (AIF), which played an essential role in the phase I chromatin condensation [39]. Analysis by electron microscopy did not reveal phase I chromatin condensation in necrotic cells dying upon MPA treatment. Furthermore, PARP-1 was found inactivated by cleavage upon the GTP depletion (Chaigne-Delalande B. and Legembre P., unpublished data). These findings support that similarly to apoptosis, necrosis has to be considered as a programmed signal, which occurs through different routes depending on the initial inducer. Characterization of these signaling pathways will be of great interest to develop new generation of anti-cancer agent that alone or in combination with an apoptotic inducer could delay or even prevent tumor relapse during chemotherapy and hence will have impact on medical treatments. In this regards, the low toxicity of MPA has already been proven in a phase I trial for the treatment of multiple myeloma [40].
